# Therapeutic Endoscopy Can Be Performed Safely in an Ambulatory Surgical Center: A Multicenter, Prospective Study

**DOI:** 10.1155/2016/7168280

**Published:** 2016-10-20

**Authors:** Shaffer R. S. Mok, Henry C. Ho, John P. Gaughan, Adam B. Elfant

**Affiliations:** Division of Gastroenterology and Liver Diseases, Department of Medicine, Cooper Medical School of Rowan University, MD Anderson Cancer Center at Cooper, Mount Laurel, NJ, USA

## Abstract

*Background.* Even amongst experienced endoscopists, endoscopic retrograde cholangiopancreatography (ERCP) and endoscopic ultrasound with fine needle aspiration (EUS-FNA) carry a potential risk for complications. These procedures are typically performed in a hospital-based endoscopy unit with general anesthesia.* Aims.* The goal of our study was to evaluate the feasibility of ERCP and EUS-FNA in an ambulatory surgical center (ASC).* Methods.* From June to November of 2014, we prospectively enrolled consecutive subjects undergoing ERCP and/or EUS-FNA in an ASC. An anesthesiologist, who was not involved in our study group, screened all subjects prior to their scheduled procedure. In order to monitor for adverse events (AE), all subjects received a telephone call at day 1 and 30 days after procedure.* Results.* 375 subjects (98 inpatients and 277 from an ASC) were enrolled. In the total population, a high proportion of subjects underwent procedures for neoplasms (21 (23.3%) inpatients versus 44 (17.1%) from an ASC) and for sphincter of Oddi dysfunction (SOD) (27 (27.5%) versus 48 (17.3%)) and had the American Society for Anesthesiologists (ASA) class ≥III (75 (76.5%) versus 140 (50.5%)) and high-risk features (17 (17.3%) versus 75 (27.1%)). Overall ERCP-related AE (10 (13.2%) versus 12 (7.5%), *p* = 0.2), pancreatitis (7 (9.2%) versus 11 (6.9%), *p* = 0.6), and hemorrhage (3.9% versus 0.6%, *p* = 0.25) were not different between inpatients and ASC subjects. There was also no difference between inpatients and ASC subjects' EUS-related AE (1 (4.5%) versus 4 (3.4%), *p* = 0.6), pancreatitis (1 (4.5%) versus 3 (2.6%), *p* = 0.2), and hemorrhage (0% versus 1 (0.9%), *p* = 0.9).* Conclusions.* ERCP and EUS can be performed in a higher risk population under the supervision of anesthesia in ASCs. Overall, the AE are equivalent between inpatients and ASC subjects.

## 1. Introduction

Endoscopic retrograde cholangiopancreatography (ERCP) and endoscopic ultrasound with fine needle aspiration (EUS-FNA) are useful techniques for a variety of pancreaticobiliary disorders. Despite their benefit, both procedures carry potential adverse events (AE), which may lead to substantial morbidity and mortality [1, 2]. In a climate of rising health costs, several authors have evaluated the safety of performing ERCP in the outpatient setting with mixed results [3–18]. Among these studies, most were retrospective and only one study evaluated EUS-FNA [3–11, 18]. Additionally, there is paucity of data to evaluate individuals undergoing ERCP/EUS-FNA in an ambulatory surgical center (ASC) and, in particular, those with cancer, elevated American Society of Anesthesiologists (ASA) class, and sphincter of Oddi dysfunction (SOD) and those determined to be at high risk [5, 19–27].

The aim of this study was to evaluate the 30-day AE of inpatient versus ASC ERCP or EUS in a multicenter, prospective cohort in high-risk subjects. We evaluated the epidemiologic and procedural risk factors for AE, as well as costs related to these procedures. Our hypothesis was that there would be no significant difference in 30-day AE when comparing the inpatient group with the ASC group.

## 2. Materials and Methods

### 2.1. Study Design

This prospective multicenter study was approved by our Institutional Review Board (IRB 14-046EX) and was performed in accordance with the Declaration of Helsinki. From June 2014 until November 2014, we prospectively enrolled consecutive subjects from three medical centers (one tertiary care center (Cooper University Hospital), two community centers (Our Lady of Lourdes Medical Center in Camden and Burlington, New Jersey) (NJ)), their subsequent ASCs (1 tertiary care endoscopy unit (Cooper University Hospital Digestive Health Institute in Mount Laurel, NJ), and 2 community-based units (Our Lady of Lourdes Medical center in Camden and Burlington, NJ)). The ASC for Cooper University Hospital was located 10 miles from its tertiary care center and the ASCs for Our Lady of Lourdes were located 1 mile away from each community hospital location. All advanced endoscopy rooms were staffed with 2 skilled endoscopy nurses and 1 skilled endoscopy technician. Informed consent was obtained from all participants for our study and no substituted consent was used.

### 2.2. Study Population

Both men and women ≥ 18 years of age were included in this study. All subjects were undergoing ERCP and/or EUS for various indications. Inpatients and ASC subjects who completed all data points and follow-up were included. We excluded pregnant women and subjects with missing data from this study.

Demographic data was then obtained from all participants and included age, sex, ethnicity, relevant comorbid conditions (i.e., cardiovascular disease (acute coronary syndrome, stroke, and systolic congestive heart failure with ejection fraction under 45%), pulmonary disease (obstructive sleep apnea, chronic obstructive pulmonary disease, etc.), cirrhosis, end-stage renal disease on dialysis), and surgical history (i.e., endoscopic interventions). A medication history was obtained to evaluate the use of anticoagulants and antiplatelet agents at the time of advanced endoscopy. All subjects received baseline liver enzymes, an amylase, and lipase to determine whether pancreatitis was present prior to the procedure [1, 28, 29].

Subjects were deemed to be at high risk by standard criterion (see supplemental appendix 1 in Supplementary Material available online at http://dx.doi.org/10.1155/2016/7168280) [1, 25]. Major criterion included one of the following: suspicion for sphincter of Oddi dysfunction (SOD) (see supplemental appendix 2), a personal history of PEP, more than 8 cannulation attempts, precut sphincterotomy, endoscopic papillary balloon dilation (EPBD) of an intact sphincter, endoscopic pancreatic duct sphincterotomy (EPS), and ampullectomy [25]. Moderate risk was defined by the following minor criterion: female sex and usage under 50 years, personal history of recurrent acute pancreatitis, pancreatic duct (PD) injection leading to opacification of acinar cells or over 3 PD injections, and PD cytology acquisition.

On the day of the examination, the indication(s) for the intervention were recorded. Then, assessment of ASA class and Mallampati score were evaluated using standard means (defined in supplemental appendix 3) [19–23]. We then elicited baseline pain scores from subjects utilizing a ten-point Likert pain scale (Appendix  4).

### 2.3. Intervention

At time zero, subjects were prospectively enrolled and given study identification using a random number generator. Prior to their procedure, an anesthesiologist determined the location of their ERCP/EUS based upon this randomization.

Three endoscopists participated in this study, all of whom had >5 years of endoscopy experience and have performed over 200 ERCPs and EUSs per year. Two postgraduate year 6 (PGY-6) fellows participated in all endoscopies performed at our tertiary care setting.

Anesthesia was administered using propofol-based monitored anesthesia care (MAC) for the duration of the procedure. No rectal indomethacin was used as prophylaxis during the study as was not the standard practice at our center during enrollment.

### 2.4. Outcomes

Presence of any AE was the primary outcome and each of the individual AE was among the secondary outcomes. AE were defined by the presence of any of the following: fever, worsening abdominal pain (based upon Likert score), gastrointestinal bleeding (GIB), infection, perforation, aspiration, need for intubation, cardiovascular arrest, acute coronary syndrome (ACS), arrhythmia, surgery, admission (if so, reason for admission, length of stay, and cost of stay), service call (if so, reason and number of calls), systemic inflammatory response syndrome (SIRS), sepsis, infection, multiorgan failure (MOF), and death (reason). Additional AE included the presence of post-ERCP pancreatitis (PEP) (see supplemental appendix 5), defined by the presence of (1) new or worsening abdominal pain that is clinically consistent with acute pancreatitis and (2) associated pancreatic enzymes elevation ≥ 3 times the upper limit of normal twenty-four hours after the procedure and (3) resultant or prolongation of existing hospitalization of ≥2 nights. Other secondary outcomes included the cost of each procedure along with subsequent AE-related costs (i.e., hospital admission and surgery) obtained using insurance data.

In order to monitor for these outcomes, data were obtained intraprocedurally and postprocedurally, as well as 1 and 30 days after endoscopy. During the procedure, hemodynamic measurements and endoscopic interventions were recorded (i.e., sphincterotomy and FNA). After their procedure, subjects were then brought to the recovery room and monitored in standard fashion. Once conscious, the ten-point pain assessment scale was again assessed. If there was a concern for AE, the subjects underwent hemodynamic monitoring and intravenous fluid (IVF) resuscitation with 1-2 liters of crystalloid and the endoscopist was then able to admit the subject to our institution if needed. If admitted, all subjects underwent basic lab work (chemistry, blood count, amylase, lipase, and liver function testing), as well as abdominal imaging if required.

To evaluate delayed complications, subjects were encouraged to return to the institution in which their procedure was performed. For comprehensive data collection, participants received a telephone call or in person encounter (when hospitalized) within 24 hours or 30 days from their procedure.

### 2.5. Statistical Methods

We determined 292 subjects would reach statistical significance. This is assuming 12% AE for ERCP and 3% for EUS with 5% risk of producing an alpha error to obtain 80% power.

Group and treatment comparisons were carried out using Fisher's exact test for categorical variables and ANOVA with contrasts for continuous variables. Outcomes were evaluated using single variable logistic regression with odds ratios and 99% confidence intervals. A *p* value of 0.01 was considered statistically significant. All analyses were carried out using SAS v9.4 software (SAS Institute, Cary, NC).

## 3. Results

### 3.1. Subjects

**Figure 1:**
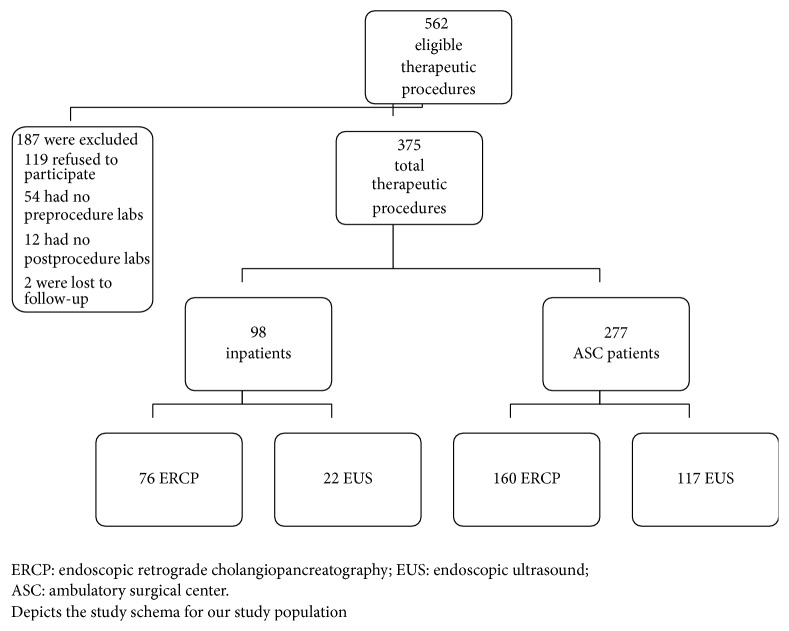
Study schema with group distribution and number/reason for exclusion in the study.

From June 2014 until November 2014, a total of 562 ERCP and EUS subjects were screened for study participation. Of those eligible for study participation, 375 agreed to participate in our study and were subsequently analyzed. Among this study sample, 98 procedures were inpatients and 277 were ASC subjects. Of these procedures, 76 were ERCP alone and 22 EUS alone were inpatients, while 160 ERCP and 117 EUS were ASC subjects (see our study schema, Figure 1). All procedures were completed and were technically successful.

Demographic and comorbid condition data did not demonstrate statistical significance between inpatients and ASC subjects (see Table 1). The mean preprocedural ASA classes were not significantly different among the inpatients as compared to the ASC population for ERCP (2.8 versus 2.3, resp.) and EUS (2.8 versus 2.6), as well as the summary of all procedures (2.8 versus 2.4, *p* = 0.24) (Table 1). Mean Mallampati scores also were not significantly different between the ERCP (1.7 versus 1.4), EUS (1.6 versus 1.5), and summary of all procedures (1.7 versus 1.5). No advanced airways were used nor was intubation performed during or prior to any of the therapeutic procedures.

Preprocedural risk factors, namely, “high-risk” components, were slightly variable among inpatients versus ASC ERCPs (22.3% versus 43.1%, resp., *p* = 0.09) and the total population (17.3% versus 27.1%, resp., *p* = 0.1) yet again did not reach statistical significance. There was no difference between high-risk features in the EUS group (0% inpatients versus 5.1% ASC subjects, *p* = 0.99). There was also no difference in moderate-risk features between the ERCP, EUS, and combined population. Table 1 demonstrated breakdown of each risk factor.

Among the inpatients versus ASC groups that underwent both ERCP and EUS, there were no significant differences in the indication. All indications can be summarized in Table 2. Evaluating indications that are considered to be at an increased risk of AE, there was no difference in procedures performed for SOD or palliation of a neoplasm. There were also a higher proportion of ASC procedures, in the total population, performed for the staging of neoplasms (11.2% versus 18.8%, *p* < 0.0001), but there was no difference in each subgroup (Table 2). Regarding findings, there were a significantly higher proportion of overall cancers (21.4% versus 15.9%, *p* < 0.0001) and pancreatic cancers (14.3% versus 13%, *p* < 0.0001) in the total inpatients population compared to ASC population. For these neoplasms, there were no significant differences in the ERCP or EUS groups.

Evaluating interventions at higher risk for AE (including needles knife sphincterortomy, manometry, ampullary biopsy, EPS, and minor duct papillotomy), we found no significant difference between inpatients and ASC subjects. There was also no significant difference in subjects who receive PD stents. All interventions are summarized in Table 2.

### 3.2. Outcomes

AE occurred in 7.2% of the study population. The overall AE rate of the total inpatient population (11.2%) was not significantly higher compared to the ASC population (5.8%, *p* = 0.11). There was also no increased risk in overall AE for ERCPs alone (13.2% inpatient versus 7.5% ASC, *p* = 0.2) or EUSs alone (4.5% versus 3.4%, *p* = 0.6).

When we evaluated each individual's procedure-related AE, no statistically significant differences in any groups or subpopulations were detected. Overall, 5.9% of subjects had PEP, among which there was no difference in the total inpatient versus ASC study population (8.2% versus 5.1%, *p* = 0.4). There was also no difference detected in the ERCP alone group (9% inpatients versus 7% ASC, *p* = 0.6). GIB occurred in 1% of the population; 3 occurred in the inpatients group, compared to two in the ASC group (*p* = 0.3). No differences were detected in the ERCP or EUS groups. No perforations or surgeries were required in our study sample and all outcomes are summarized in Table 3.

When evaluating 30-day mortality (3.2% overall), there was no significant difference among the total populations (9% versus 1%), ERCP (9% versus 1%), and EUS (5% versus 2%) (Table 3). Overall, there were 8 subjects who died in the inpatients group and 3 in the ASC group (9.2% versus 1.1%, *p* = 0.09). The time from procedure until death for the inpatients was 15.4 days (12, 18, 23, 30, 10, 5, and 25 days), compared with 20.7 days in ASC subjects (28, 25, and 27 days). All deaths, which occurred in this study, were the result of cancer-related mortality or while in a hospice and not from a procedure-related AE. Of the inpatient population, 2 of the cancer-related deaths were from nongastrointestinal septic shock, while another also developed respiratory compromise and cardiac arrest within 30 days. Among the ASC population, one subject developed combined septic/cardiogenic shock after surgery for their neoplasm. Another ASC subject developed respiratory failure and subsequent cardiac arrest within 30 days after having their procedures for neoplastic encasement. This subject had pancreatitis both before and after procedure.

We found no significant difference in service calls for any reason (Table 3). There was also no difference in the number of readmissions, ED visits, hospitalizations, or urgent care visits among any of the groups (Table 3). The patients undergoing readmission were not different in the ERCP group (11% versus 6), EUS group (5% versus 9%), or summary groups (19% versus 7%). Mean LOS was significantly longer in the total inpatient study population (8.7 days versus 0.8 days, *p* < 0.0001) and inpatient ERCP versus ASC groups (9.3 days versus 0.6 days, *p* < 0.0001, resp.). No significant difference in LOS was detected in the EUS group (Table 3).

Finally, mean procedural and total medical cost was evaluated. Mean procedural cost was significantly higher in the inpatient total population compared with the ASC population ($482.30 versus $423.20, *p* < 0.0001). No difference in cost was seen in the ERCP or EUS study groups. When evaluating mean total medical cost, both the total ($17,815.70 versus $2,026.90, *p* < 0.0001) and ERCP ($19,022.90 versus $1,574.30, *p* < 0.0001) inpatient populations were significantly larger than their ASC counterparts. The EUS group was not statistically different between the inpatient and ASC populations (Table 3).

## 4. Discussion

In this prospective, multicenter, observational study, we demonstrated no difference in overall or individual AE for subjects undergoing inpatient versus ASC advanced endoscopy. Our study population demonstrated a low AE rate, even with an increased incidence of high-risk procedural features and proportion with an ASA class ≥3.

At present, the American Society for Gastrointestinal Endoscopy (ASGE) has released two documents regarding quality indicators in therapeutic endoscopy [30, 31]. Within these documents are established rates of AE after advanced endoscopic procedures. Generally, it is estimated that ERCP-related AE occur in 5–8% of procedures, with relative mortality of 0.5–2% [1, 2, 7, 16, 30, 31]. Additionally, a similar risk of 0.5–2.9% has been estimated for EUS-FNA [2, 18, 31]. All in all, several prospective studies have been performed and a subsequent review of this topic yielded an overall vision of safety for ERCP in the ASC setting [3–18]. Despite this available evidence, there still exists the question of whether ASC therapeutic endoscopy is safe in a population at higher risk for AE or an ASA class ≥3.

Overall there is paucity of data regarding the safety of therapeutic endoscopy procedures in the ASC cancer population. Composite data from prior studies yielded low proportions of ampullary and pancreatic carcinomas when compared with our population [18]. Mehta et al.'s study did include ASC EUS which were performed for upper GI lesions and for luminal malignancies [7]. In addition to this study, Cvetkovski et al. evaluated this unique population in a retrospective chart review with low AE rates but with no data on postprocedural medical care or cost [5]. Yet the percentage of cancers in our population does appear to be larger than prior studies (17%, other studies, versus 18.7%, our study) [3–18].

Other than cancer subjects, another unique determination in our study is evaluation of AE in the ASC setting using this “higher risk” population. Freeman et al., among others, have evaluated various risk factors, which served to increase the risk of PEP, along with other AE after ERCP [1, 25]. Most other studies evaluating ASC therapeutic endoscopy occurred prior to the identification of high-risk features for post-ERCP AE [3–18]. Yet dissection of these studies included a minimal percentage of subject characteristics determined to be high/moderate risk. Overall, our study was composed of a large proportion of high/moderate-risk subjects (24.5% and 25.1%, resp.). Of this high-risk population, we had 20% that underwent advanced endoscopic procedures for SOD (27% of ERCPs). The most recent study, evaluating ASC therapeutic endoscopy, was published by Rábago et al., with 3% of individuals undergoing ERCP for SOD, and in Mehta et al.'s study, the percentage was 14% [7, 13]. The aforementioned Mehta et al.'s study had 18.9% of ERCP performed for SOD, which is the highest among the studies performed for ASC therapeutic endoscopy [7]. Despite this, the average percentage of subjects included in prior studies for ASC therapeutic endoscopy with “higher risk features” was 9% of ASC subjects and 1% of inpatients with SOD, far lower than our population.

In addition to high-risk procedural factors, our study population did include a large proportion of subjects who had ASA classes greater than or equal to III (57.3% overall), determined to be high-risk from an anesthesia perspective. The ASA along with the ASGE has also expressed usage of ASA classes prior to endoscopic procedures [19–23, 32]. Within this document is the determination of ASA classes ≥III as high risk and subsequent need for anesthesia monitoring during endoscopy. This sentiment was examined by Coté et al. demonstrating significant risk for ASA scoring ≥3 predicting the need for airway maneuvers during advanced endoscopic procedures [33]. Other retrospective analyses have evaluated ASA scoring as a risk for serious AE in the setting of therapeutic endoscopy [34, 35]. As a result, most prior studies evaluating ASC therapeutic endoscopy either included a minimal number of individuals with ASA classes ≥III or excluded them entirely [3–18]. Among these studies, the highest percentages of ASA classes ≥III were seen in Mahnke et al. (17.5%) and Hui et al. (33.2%), with Mahnke et al. being the only prospective investigation [14, 18]. Again, these prior series have demonstrated lower incidence of ASA ≥3 when compared to our population.

At our institution, we utilize propofol-based anesthesia under the guidance of an anesthesiologist/Certified Registered Nurse Anesthetists (CRNA) for all endoscopic procedures. Using this care model, with no statistical difference in the mean ASA class in the inpatient population compared with the ASC population (2.8 inpatients versus 2.4 ASC subjects, *p* = 0.24), our study found no increased risk of overall postprocedural AE, PEP, hemorrhage, mortality, and so forth between these sample groups. Additionally, an elevated ASA class also led to additional 2.1 days in the hospital when admission was needed, but there was no increased risk for ED visit, hospitalization, urgent care visits, service calls, or readmissions. Thus, performance of ASC ERCP, EUS, or combined procedures in those with high-risk features, interventions, and an ASA class ≥III led to no increase in AE and medical disposition with a mild increase in LOS among inpatients.

Potential weakness could have included our cost analysis for procedures that may have been different between inpatients and ASC subjects because of the instruments utilized between procedures. However, standardized tools were utilized for all cases. Another potential risk may have been the large number of variables examined, which led to our statistical cut-off being *p* < 0.01 and not *p* < 0.05. This correction was used to eliminate errors from random chance and may have excluded some relevant findings. Points of strength of this paper included the prospective nature for which this study was performed and randomization of procedure location based upon a random number generator. We also methodically obtained postprocedure labs in all subjects and data with rigorous follow-up. To evaluate our study parameters, we also evaluated subjects in tertiary care centers as well as community centers; thus our information applied to both practice settings. Another point of strength was validating our data in a unique cohort of subjects described as having high-risk procedural and preprocedural risks, higher ASA classes, and a larger proportion of subjects with cancer. This allowed for more generalizable interpretation of our data. In future study, we hope to validate the safety of ASC AE in combined ERCP/EUS and therapeutic EUS and by incorporating prophylactic measures such as intravenous fluid strategy and rectal indomethacin.

In an age where healthcare costs continue to rise, the feasibility of performing both ERCP and EUS safely in the ASC setting has become of paramount importance. Also, with the publication of value-based metrics set forth by the ASGE, it has been integral for endoscopists to perform quality therapeutic procedures in a manner safe for subjects. In this prospective multicenter study, with 30-day follow-up, we determined that ERCP and EUS are safe and cost-effective procedures in the high-risk ASC population.

## Supplementary Material

All information contained in supplemental appendix 1 demonstrates the risk factors which make individuals at high risk for post-ERCP pancreatitis.Information in supplemental appendix 2 incudes the Rome criterion for sphincter of Oddi dysfunction. Supplement 3, includes the criterion for describing ASA classification in tabular form. Supplement 4 is the standard 10 point Likert pain score.

## Figures and Tables

**Table 1:** Demographic and risk data for inpatient versus ASC ERCP, EUS, and the total population.

	ERCP				EUS				Total			
	IN	ASC	ALL	*p* ^*∗*^ (95% CI)	IN	ASC	All	*p* ^*∗*^ (95% CI)	IN	ASC	ALL	*p* ^*∗*^ (95% CI)
Total cases	76	160	236		22	117	139		98	277	375	
Mean age (years)	61.8	60.2	62	0.54	64.2	67.6	63.5	0.62	62.7	63.8	63.5	0.85
Sex												
Male	31 (41%)	46 (29%)	77 (33%)	0.17	18 (82%)	56 (48%)	74 (53%)	0.13	49 (50%)	102 (37%)	151 (40%)	0.1
Female	45 (59%)	114 (71%)	159 (67%)		4 (18%)	61 (52%)	65 (47%)		49 (50%)	175 (63%)	224 (60%)	
Comorbid conditions												
Cardiovascular disease	35 (46%)	55 (34%)	90 (38%)	0.12	4 (18%)	57 (49%)	61 (44%)	0.22	39 (40%)	112 (40%)	151 (40%)	0.27
Pulmonary disease	17 (22%)	18 (11%)	35 (15%)	0.04	2 (9%)	17 (15%)	19 (14%)	0.53	19 (19%)	35 (13%)	54 (14%)	0.97
ESRD on dialysis	12 (16%)	27 (17%)	39 (17%)	0.70	1 (5%)	31 (27%)	32 (23%)	0.28	16 (16%)	62 (22%)	78 (21%)	0.03
Cirrhosis	5 (7%)	7 (4%)	12 (5%)	0.53	0	5 (4%)	5 (4%)	0.99	5 (5%)	13 (5%)	18 (5%)	0.98
Medications												
Anticoagulation	3 (4%)	6 (4%)	9 (4%)	1.00	1 (5%)	4 (3%)	5 (4%)	0.28	4 (4%)	10 (4%)	14 (4%)	0.55
Antiplatelet agent	16 (21%)	23 (14%)	39 (17%)	0.25	1 (5%)	25 (2%)	26 (19%)	0.43	17 (17%)	48 (17%)	65 (17%)	0.07
Endoscopic risk factors												
High risk features	17 (22%)	69 (43%)	86 (36%)	0.09	0	6 (5%)	6 (4%)	0.99	17 (17%)	75 (27%)	92 (25%)	0.1
History of SOD	4 (5%)	19 (12%)	23 (10%)	0.09	0	6 (5%)	6 (4%)	1.00	4 (4%)	25 (7%)	29 (6%)	0.72
History of post-ERCP pancreatitis	0	0	0	1.00	0	0	0	1.00	0	0	0	1.00
Pancreatic sphincterotomy	7 (9%)	37 (23%)	44 (19%)	0.03	NA			1.00	7 (7%)	37 (13%)	44 (12%)	0.01
Precut sphincterotomy	1 (1%)	1 (1%)	2 (1%)	0.62	NA			1.00	1 (1%)	1 (1%)	2 (1%)	1.00
>8 cannulation attempts	0	0	0	1.00	NA			1.00	0	0	0	1.00
Pneumatic dilation of an intact biliary sphincter	5 (7%)	9 (6%)	14 (6%)	0.84	NA			1.00	5 (5%)	9 (3%)	14 (4%)	1.00
Ampullectomy	0	3 (2%)	3 (1%)	0.95	NA			1.00	0	3 (1.1%)	3 (0.8%)	1.00
Moderate risk features	19 (25%)	68 (43%)	87 (37%)	0.10	0	7 (6%)	7 (5%)	0.98	19 (19%)	75 (27%)	94 (25%)	0.02
Age < 50 and female	9 (12%)	26 (16%)	35 (15%)	0.29	0	3 (3%)	3 (2%)	0.99	9 (9%)	29 (11%)	38 (10%)	0.09
History of recurrent pancreatitis	6 (8%)	7 (4%)	13 (6%)	0.38	0	4 (3%)	4 (3%)	0.99	6 (6%)	11 (4%)	17 (5%)	0.93
>3 injections to PD, 1 to tail	1 (1%)	11 (7%)	12 (5%)	1.00	NA			1.00	1 (1%)	11 (4%)	12 (3%)	1.00
Excessive injection PD contrast, leading to acini	0	0	0	1.00	NA			1.00	0	0	0	1.00
Acquisition of cytology from PD using brush	3 (4%)	5 (3%)	8 (3%)	0.30	NA			1.00	3 (3%)	5 (2%)	8 (2%)	0.30
Anesthesia risk												
Mean Mallampati score	1.7	1.4	1.5	0.09	1.6	1.5	1.5	0.93	1.7	1.5	1.5	0.02
Mean ASA score	2.8	2.3	2.5	0.12	2.8	2.6	2.6	0.38	2.8	2.4	2.5	0.24
1	2 (3%)	17 (11%)	19 (8%)		0	1 (1%)	1 (1%)		2 (2%)	18 (7%)	20 (5%)	
2	17 (22%)	74 (46%)	91 (39%)		4 (18%)	45 (39%)	49 (35%)		21 (21%)	119 (43%)	140 (37%)	
3	52 (68%)	69 (43%)	121 (51%)		18 (82%)	71 (61%)	89 (64%)		70 (71%)	140 (51%)	210 (56%)	
4	5 (7%)	0	5 (2%)		0	0	0		5 (5%)	0	5 (1%)	

ERCP: endoscopic retrograde cholangiopancreatography; EUS: endoscopic ultrasound; IN: inpatients; ASC: ambulatory surgical center patients; ALL: inpatients + ASC patients; CI: confidence interval; ESRD: end-stage renal disease; SOD: sphincter of Oddi dysfunction; PD: pancreatic duct; ASA: American society of anesthesia class.

^*∗*^Note that *p* < 0.01 is significant.

**Table 2:** Indications, findings, and interventions for inpatient versus ASC ERCP, EUS, and the total population.

	ERCP				EUS				Total			
	IN	ASC	ALL	*p* ^*∗*^ (95% CI)	IN	ASC	ALL	*p* ^*∗*^ (95% CI)	IN	ASC	ALL	*p* ^*∗*^ (95% CI)
*Indications*												
Biliary												
Obstructive jaundice	39 (51%)	37 (23%)	76 (32%)	**0.0005**	10 (46%)	8 (7%)	18 (13%)	0.07	49 (50%)	45 (16%)	94 (25%)	**<0.0001**
SOD	24 (32%)	40 (25%)	64 (27%)	0.02	3 (14%)	8 (7%)	11 (8%)	0.98	27 (28%)	48 (17%)	75 (20%)	0.02
Dilated biliary ductal system	31 (41%)	25 (16%)	56 (24%)	**<** **0.0001**	4 (18%)	5 (4%)	9 (7%)	0.94	35 (36%)	30 (11%)	65 (17%)	**0.003**
Choledocholithiasis	19 (25%)	22 (14%)	41 (17%)	0.046	0	0	0	1.00	19 (19%)	22 (8%)	41 (11%)	0.55
Elevated LFT	17 (20%)	5 (3%)	22 (9%)	**0.0002**	3 (14%)	1 (1%)	4 (3%)	1.00	20 (20%)	6 (2%)	26 (7%)	**0.004**
Cholangitis	9 (12%)	0	9 (4%)	0.92	0	0	0	1.00	9 (9%)	10 (3%)	9 (2%)	1.00
Biliary stricture	3 (4%)	8 (5%)	11 (5%)	0.37	1 (5%)	1 (1%)	2 (1%)	0.99	4 (4%)	9 (3%)	13 (4%)	0.62
Stent extraction	9 (12%)	28 (18%)	37 (16%)	0.12	1 (5%)	0	1 (1%)	0.99	10 (10%)	28 (10%)	38 (10%)	0.03
Bile leak	10 (13%)	14 (9%)	24 (10%)	0.53	1 (5%)	0	1 (1%)	0.98	11 (11%)	14 (5%)	25 (7%)	0.35
Pancreatic												
Pancreatic mass	8 (11%)	10 (6%)	18 (8%)	0.09	6 (27%)	26 (22%)	32 (23%)	0.19	14 (14%)	36 (13%)	50 (13%)	**<0.0001**
Pancreatic cyst	0	6 (4%)	6 (3%)	0.96	1 (5%)	38 (33%)	39 (28%)	0.22	1 (1%)	44 (16%)	45 (12%)	**<0.0001**
Chronic pancreatitis	2 (3%)	16 (10%)	18 (8%)	0.11	1 (5%)	8 (7%)	9 (7%)	0.37	3 (3%)	24 (9%)	27 (7%)	0.48
Gall stone pancreatitis	11 (15%)	1 (1%)	12 (5%)	**0.003**	3 (14%)	3 (3%)	6 (3%)	0.02	14 (14%)	4 (1%)	18 (5%)	**0.009**
Pancreatic divisum	1 (1%)	10 (6%)	11 (5%)	0.11	0	2 (2%)	2 (1%)	0.99	1 (1%)	14 (5%)	15 (4%)	0.44
Neoplastic												
Any neoplasm	11 (15%)	12 (8%)	23 (10%)	0.10	3 (14%)	29 (25%)	32 (23%)	0.61	21 (21%)	44 (16%)	65 (17%)	**<0.0001**
Palliation neoplasm	5 (7%)	1 (1%)	6 (3%)	0.06	2 (9%)	0	2 (1%)	1.00	7 (7%)	1 (1%)	8 (2%)	0.50
Staging of neoplasm	6 (8%)	6 (4%)	12 (5%)	0.68	5 (23%)	46 (39%)	51 (37%)	0.98	11 (11%)	52 (19%)	63 (17%)	**<0.0001**
Generalized												
Chronic abdominal pain	8 (11%)	53 (33%)	61 (26%)	**0.0004**	0	6 (5%)	6 (3%)	0.99	8 (8%)	59 (21%)	67 (18%)	**<0.0001**
*Intervention*												
ERCP												
Biliary												
EBS	57 (75%)	108 (68%)	165 (70%)	**<0.0001**	NA			1.00	57 (58%)	108 (39%)	165 (44%)	**<0.0001**
Needle knife	1 (1%)	1 (1%)	2 (1%)	1.00	NA			1.00	1 (1%)	1 (1%)	2 (1%)	1.00
SEMS	12 (16%)	5 (3%)	17 (8%)	0.06	NA			1.00	12 (12%)	5 (2%)	17 (5%)	0.06
Plastic stent	23 (30%)	18 (11%)	41 (17%)	**0.003**	NA			1.00	23 (24%)	18 (7%)	41 (11%)	**0.003**
Cytology	11 (15%)	22 (14%)	33 (14%)	**0.0002**	NA			1.00	11 (1%)	22 (8%)	33 (9%)	**0.0002**
Cholangioscopy	4 (5%)	11 (7%)	15 (11%)	0.40	NA			1.00	4 (4%)	11 (4%)	15 (4%)	0.40
Manometry	2 (3%)	18 (11%)	20 (9%)	0.47	NA			1.00	2 (2%)	18 (7%)	20 (5%)	0.47
Pancreatic												
EPS	6 (8%)	30 (19%)	36 (15%)	0.03	NA			1.00	6 (6%)	30 (11%)	36 (10%)	0.03
Minor duct papillotomy	1 (1%)	8 (5%)	9 (4%)	0.92	NA			1.00	1 (1%)	8 (3%)	9 (2%)	0.92
PD Stent	21 (28%)	44 (28%)	65 (28%)	1.00	NA			1.00	21 (21%)	44 (16%)	65 (17%)	0.60
Ampullary biopsy	2 (3%)	13 (8%)	15 (6%)	0.13	0	1 (1%)	1 (1%)	1.00	3 (3%)	14 (5%)	17 (5%)	0.81
EUS												
FNA	NA			1.00	12 (55%)	77 (66%)	89 (64%)	**<0.0001**	12 (12%)	77 (28%)	89 (24%)	**<0.0001**

ERCP: endoscopic retrograde cholangiopancreatography; EUS: endoscopic ultrasound; IN: inpatients; ASC: ambulatory surgical center patients; ALL: inpatients + ASC patients; GI: gastrointestinal; LFT: liver function tests; SOD: sphincter of Oddi dysfunction; GIST: gastrointestinal stromal tumor; IPMN: intraductal papillary mucinous neoplasm; NE: neuroendocrine; EBS: endoscopic biliary sphincterotomy; EPS: endoscopic pancreatic sphincterotomy; SEMS: self-expanding metal stent; FNA: fine needle aspiration.

^*∗*^Note that *p* < 0.01 is significant.

**Table 3:** Outcomes and cost for inpatient versus ASC ERCP, EUS, and the total population.

	ERCP				EUS				Total			
	IN	ASC	ALL	*p* ^*∗*^ (95% CI)	IN	ASC	ALL	*p* ^*∗*^ (95% CI)	IN	ASC	ALL	*p* ^*∗*^ (95% CI)
*Outcomes*												
Postprocedural complications	10 (13%)	12 (8%)	18 (8%)	0.20	1 (5%)	4 (3%)	5 (4%)	0.60	11 (11%)	16 (6%)	27 (7%)	0.11
Pancreatitis	7 (9%)	11 (7%)	18 (8%)	0.60	1 (5%)	3 (3%)	4 (3%)	0.20	8 (8%)	14 (5%)	22 (6%)	0.43
Hemorrhage	3 (4%)	1 (1%)	4 (2%)	0.25	0	1 (1%)	1 (1%)	0.98	3 (3%)	2 (0.7%)	5 (1%)	0.92
Perforation	0	0	0	1.00	0	0	0	1.00	0	0	0	1.00

*Mortality*												
All-cause	7 (9%)^a,b^	1 (1%)^c^	8 (3%)	0.01	1 (5%)	2 (2%)^d^	3 (2%)	0.12	9 (9%)	3 (1%)	12 (3%)	0.09

*Service call*												
Fevers	7 (9%)	2 (1%)	9 (4%)	0.02	2 (9%)	2 (2%)	4 (3%)	1.00	9 (9%)	4 (1%)	13 (4%)	0.047
Nausea/vomiting	8 (11%)	17 (11%)	25 (11%)	0.87	0	7 (6%)	7 (5%)	0.98	8 (8%)	24 (9%)	32 (9%)	0.79
Abdominal pain	16 (21%)	37 (23%)	53 (23%)	0.41	2 (9%)	11 (9%)	13 (9%)	0.98	18 (18%)	48 (17%)	66 (18%)	0.13

*Medical care*												
ED	2 (3%)	5 (3%)	7 (3%)	0.60	0	2 (2%)	2 (%)	0.94	2 (2%)	7 (3%)	9 (2%)	0.70
Urgent care^e^	1 (1%)	2 (1%)	3 (1%)	1.00	0	0	0	1.00	1 (1%)	2 (1%)	3 (1%)	0.90
Hospitalization^f^	1 (1%)	4 (3%)	5 (2%)	0.32	0	2 (2%)	2 (1%)	0.94	1 (1%)	6 (2%)	7 (2%)	0.34
LOS	9.3	0.6	3.6	**<0.0001**	4.4	0.7	2.8	0.21	8.7	0.8	2.8	**<0.0001**
Readmission	8 (11%)	10 (6%)	18 (8%)	0.04	1 (5%)	10 (9%)	11 (8%)	0.80	9 (19%)	20 (7%)	29 (8%)	0.17

*Cost*												
Procedure	$489.60	$474.30	$490.70	0.12	$339.10	$292.20	$438.40	0.35	$482.30	$423.20	$438.40	**<0.0001**
Total	$19,022.90	$1,574.30	$7,662.80	**<0.0001**	$9,196.30	$1,668.90	$6,082.20	0.21	$17,815.70	$2,026.90	$6,082.20	**<0.0001**

ERCP: endoscopic retrograde cholangiopancreatography; EUS: endoscopic ultrasound; IN: inpatients; ASC: ambulatory surgical center patients; ALL: inpatients + ASC patients; ARDS: acute respiratory distress syndrome; CI: confidence interval; SIRS: systemic inflammatory response syndrome; MOF: multiple organ failure; MI: myocardial infarction; ED: emergency department; LOS: length of stay.

^*∗*^Note that *p* < 0.01 is significant.

^a^Inpatient that suffered cardiac arrest or respiratory failure was found to have septic shock and mild pancreatitis on day 21 after ERCP.

^b^A second inpatient after cancer surgery died of septic shock.

^c^ASC admitted on day 28 with pancreatitis or respiratory failure was DNR/DNI but family desired to be made hospice.

^d^ASC admitted with septic/cardiogenic shock after surgery for cancer.

^e^Note that no urgent care visits were hospitalized.

^f^Note that all hospitalizations were sent from a call to the service or directly from the endoscopy unit to the ED.

## Abbreviations

ERCP: Endoscopic retrograde cholangiopancreatography
EUS: Endoscopic ultrasound
EUS-FNA: Endoscopic ultrasound with fine needle aspiration
ASC: Ambulatory surgical center
SOD: Sphincter of Oddi dysfunction
ASA: American Society for Anesthesiologists
NJ: New Jersey
MAC: Monitored anesthesia care
PGY: Postgraduate year
BSC: Boston Scientific Corporation, Incorporated
ACEI: Angiotensin converting enzymes inhibitors
ARB: Angiotensin II receptor blocker
IVF: Intravenous fluids
ED: Emergency department
PEP: Post-ERCP pancreatitis
GIB: Gastrointestinal bleeding
ACS: Acute coronary syndrome
SIRS: Systemic inflammatory response syndrome
MOF: Multiple organ failure
ESRD: End-stage renal disease
LOS: Length of stay
ANOVA: Analysis of variance
SAS: Statistical analysis system
OR: Odds ratio
EBS: Endoscopic biliary sphincterotomy
EPS: Endoscopic pancreatic sphincterotomy
ASGE: American Society for Gastrointestinal Endoscopy
CRNA: Certified Registered Nurse Anesthetists.
