# Supplementary material for: Therapeutic Endoscopy Can Be Performed Safely in an Ambulatory Surgical Center: A Multicenter, Prospective Study
Source: Diagn Ther Endosc. 2016 Oct 20;2016:7168280. doi: 10.1155/2016/7168280 (PMC5093287; doi:10.1155/2016/7168280)
Supplement: Supplementary file 1 — All information contained in supplemental appendix 1 demonstrates the risk factors which make individuals at high risk for post-ERCP pancreatitis.Information in supplemental appendix 2 incudes the Rome criterion for sphincter of Oddi dysfunction. Supplement 3, includes the criterion for describing ASA classification in tabular form. Supplement 4 is the standard 10 point Likert pain score. [file 7168280.f1.docx]

**APPENDIX 1**

**Definition of High Risk Patient Characteristics^1-5^**

**Any one of the following**

**Historical characteristics**

1. Sphincter of Oddi dysfunction (APPENDIX 2)
2. A history of post-ERCP pancreatitis (APPENDIX 1)

**ERCP characteristics**

1. Pancreatic sphincterotomy
2. Precut sphincterotomy (a procedure performed to facilitate biliary access when standard cannulation techniques are unsuccessful)
3. More than eight cannulation attempts (as determined by the endoscopist)
4. Pneumatic dilatation of an intact biliary sphincter
5. Ampullectomy.

**Or two or more of the following**

**Historical characteristics**

1. Age of less than 50 years and female sex
2. History of recurrent pancreatitis (≥2 episodes)

**ERCP characteristics**

1. Three or more injections of contrast agent into the pancreatic duct with at least one injection to the tail of the pancreas
2. Excessive injection of contrast agent into the pancreatic duct resulting in opacification of pancreatic acini
3. Acquisition of a cytologic specimen from the pancreatic duct with the use of a brush
4. Anderson MA, Fisher L, Jain R, et al. American Society for Gastrointestinal Endoscopy Practice Guidelines: Complications of ERCP. Gastrointest Endosco. 2012;75(3):467-73.
5. **Freeman ML, Nelson DB, Sherman S, et al.** Complications of endoscopic biliary sphincterotomy. N Engl J Med. 1996;335(13):909-18.
6. Yaghoobi M, Rolland S, Waschke KA, et al. Meta-analysis: rectal indomethacin for the prevention of post-ERCP pancreatitis. Aliment Pharmacol Ther. 2013;38(9):995-1001.
7. Elmunzer BJ, Waljee AK, Elta GH, et al. A meta-analysis of rectal NSAIDs in the prevention of post-ERCP pancreatitis. Gut. 2008;57:1262-1267.
8. Murray B, Carter R, Imrie C, et al. Diclofenac reduces the incidence of acute pancreatitis after endoscopic retrograde cholangiopancreatography. Gastroenterology. 2003;124:1786–91

**APPENDIX 2**

**Definition of Sphincter of Oddi Dysfunction (SOD)**

**SOD Pain is defined^1,2^**

- Pain in the upper part of the abdomen and/or the right upper quadrant of the abdomen
- Episodes of pain must last at least 30 minutes
- The symptoms must be recurrent, and occur at differing intervals
- The pain must incrementally increase to a "steady level"
- The pain must be severe enough the patient's daily activities are affected, or that the patient must attend the emergency department
- The pain must not be relieved by any of bowel movements, change in posture, or antacids; and,
- Other structural disorders that could explain the symptoms must be excluded.

**Functional Biliary SOD Disorder^1,2^**

- Type I biliary SOD: biliary-type abdominal pain, with all of altered liver enzymes on blood testing, dilated biliary ducts on ultrasound or ERCP (>9mm) , and delayed bile clearance on HIDA^a^ scan (<35%).
- Type II biliary SOD: biliary-type abdominal pain associated with one or two of the following: altered liver enzymes on blood testing, dilated biliary ducts on imaging tests (>9mm), and delayed bile clearance on HIDA scan (<35%).
- Type III biliary SOD: biliary-type abdominal pain with none of the following: altered liver enzymes on blood testing, dilated biliary ducts on imaging tests (>9mm), and delayed bile clearance on HIDA scan (<35%).

**Functional Pancreatic SOD Disorder^1,2^**

- Individuals are classified as having a functional pancreatic sphincter of Oddi disorder if the above criteria are met, and if the testing of pancreatic enzymes (amylase and lipase) is abnormal

^a^HIDA = Hepatobiliary iminodiacetic acid

1. Rome Foundation. Rome III Diagnostic Criteria for Functional Gastrointestinal Disorders. Rome Foundation. Retrieved August, 2014.

2. Prajapati DN & Hogan WJ. Sphincter of Oddi dysfunction and other functional biliary disorders: Evaluation and treatment. Gastro clin of North America. 2003;32(2):601–18.

**Appendix 3.1**

**American Society of Anesthesiologists (ASA)**

**Physical status classification system**

| **Class** | **Original** |  |
| --- | --- | --- |
| 1 | No organic pathology or patients in whom the pathological process is localized and does not cause any systemic disturbance or abnormality.  Examples: This includes patients suffering with fractures unless shock, blood loss, emboli or systemic signs of injury are present in an individual who would otherwise fall in Class 1. It includes congenital deformities unless they are causing systemic disturbance. Infections that are localized and do not cause fever, many osseous deformities, and uncomplicated hernias are included. Any type of operation may fall in this class since only the patient's physical condition is considered. |  |
| 2 | A moderate but definite systemic disturbance, caused either by the condition that is to be treated or surgical intervention or which is caused by other existing pathological processes, forms this group.  Examples: Mild diabetes. Functional capacity I or IIa. Psychotic patients unable to care for themselves. Mild acidosis. Anemia moderate. Septic or acute pharyngitis. Chronic sinusitis with postnasal discharge. Acute sinusitis. Minor or superficial infections that cause a systemic reaction. (If there is no systemic reaction, fever, malaise, leukocytosis, etc., aid in classifying.) Nontoxic adenoma of thyroid that causes but partial respiratory obstruction. Mild thyrotoxicosis. Acute osteomyelitis (early). Chronic osteomyelitis. Pulmonary tuberculosis with involvement of pulmonary tissue insufficient to embarrass activity and without other symptoms. |  |
| 3 | Severe systemic disturbance from any cause or causes. It is not possible to state an absolute measure of severity, as this is a matter of clinical judgment. The following examples are given as suggestions to help demonstrate the difference between this class and Class 2.  Examples: Complicated or severe diabetes. Functional capacity IIb. Combinations of heart disease and respiratory disease or others that impair normal functions severely. Complete intestinal obstruction that has existed long enough to cause serious physiological disturbance. Pulmonary tuberculosis that, because of the extent of the lesion or treatment, has induced vital capacity sufficiently to cause tachycardia or dyspnea. Patients debilitated by prolonged illness with weakness of all or several systems. Severe trauma from accident resulting in shock, which may be improved by treatment. Pulmonary abscess. |  |
| 4 | Extreme systemic disorders which have already become an eminent threat to life regardless of the type of treatment. Because of their duration or nature there has already been damage to the organism that is irreversible. This class is intended to include only patients that are in an extremely poor physical state. There may not be much occasion to use this classification, but it should serve a purpose in separating the patient in very poor condition from others.  Examples: Functional capacity III -(Cardiac Decompensation). Severe trauma with irreparable damage. Complete intestinal obstruction of long duration in a patient who is already debilitated. A combination of cardiovascular-renal disease with marked renal impairment. Patients who must have anesthesia to arrest a secondary hemorrhage where the patient is in poor condition associated with marked loss of blood. *Emergency Surgery:* An emergency operation is arbitrarily defined as a surgical procedure which, in the surgeon's opinion, should be performed without delay. |  |
| 5 | Emergencies that would otherwise be graded in Class 1 or Class 2. |  |
| 6 | Emergencies that would otherwise be graded as Class 3 or Class 4. |  |

1. Saklad M. Grading of patients for surgical procedures. Anesthesiology 1941;2:281-4.

2. ASA Relative Value Guide 2002, American Society of Anesthesiologists, page xii, Code 99140.

3. ASA Physical Status Classification System. American Society of Anesthesiologists. Retrieved 2013.

**Appendix 3.2**

**Malampati Score^4^**

**Mallampati Scoring**

- Class I: Soft palate, uvula, fauces, pillars visible.
- Class II: Soft palate, uvula, fauces visible.
- Class III: Soft palate, base of uvula visible.
- Class IV: Only hard palate visible

[
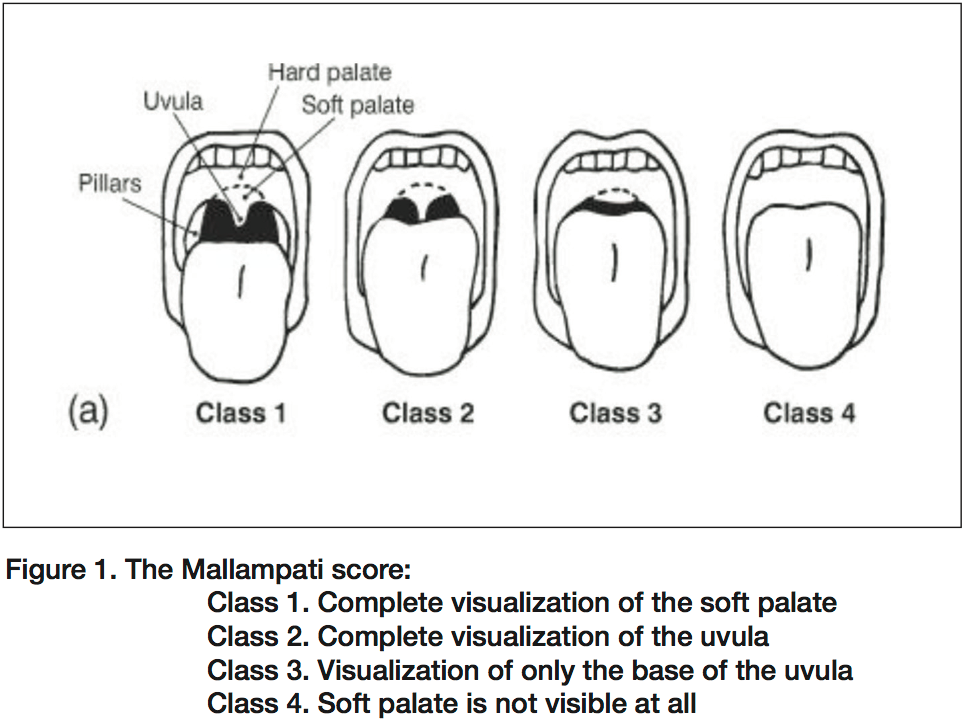
](http://www.google.com/url?sa=i&rct=j&q=&esrc=s&source=images&cd=&docid=A2lTnAUho-_3OM&tbnid=PolQSGVhDugESM:&ved=0CAUQjRw&url=http://www.cathlabdigest.com/articles/Ask-Clinical-Instructor-20&ei=Wwc_U7HgKsuksQT77oAI&bvm=bv.64125504,d.dmQ&psig=AFQjCNF-GH74zvnLertEbzUfVHTWoq4CDQ&ust=1396725940446565)

4. Mallampati, SR; Gatt, SP; Gugino, LD; Desai, SP; Waraksa, B; Freiberger, D; Liu, PL (1985 Jul). "A clinical sign to predict difficult tracheal intubation: a prospective study.". *Canadian Anaesthetists' Society journal* 32(4): 429–34.

**APPENDIX 4**

**Likert Pain Scale**

[
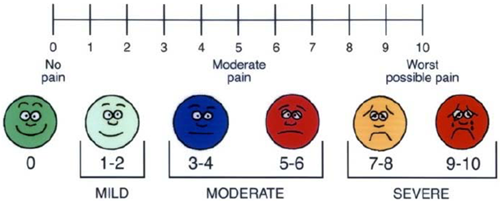
](http://www.google.com/url?sa=i&rct=j&q=&esrc=s&source=images&cd=&docid=2AdhJ2X7z9wSBM&tbnid=WQ8L5VPboR6hLM:&ved=0CAUQjRw&url=http://www.paradigmcorp.com/blog/?tag=oswestry-low-back-pain-scale&ei=0gaKU4T4OpXTsAS94oGwBw&bvm=bv.67720277,d.aWw&psig=AFQjCNHwvHePtW5i0LLsWBLNNkKH4jidxQ&ust=1401641001184695)

**APPENDIX 5**

**The Definition of Post-ERCP pancreatitis and Acute Pancreatitis**

**Post-ERCP Pancreatitis^1,2^**

(1) new or worsened abdominal pain

(2) new or prolongation of hospitalization for at least 2 days, and

(3) serum amylase 3 times or more the upper limit of normal, measured more than 24 hours after the procedure

**Acute Pancreatitis^3^**

The diagnosis of AP is most often established by the presence of 2 of the 3 following criteria:

1. abdominal pain consistent with the disease
2. serum amylase and / or lipase greater than three times the upper limit of normal, and / or
3. characteristic findings from abdominal imaging

1. Cotton PB, Lehman G, Vennes J, et al. Endoscopic sphincterotomy complications and their management: an attempt at consensus. Gastrointest Endosc. 1991;37(3):383.

2. Anderson MA, Fisher L, Jain R, et al. American Society for Gastrointestinal Endoscopy Practice Guidelines: Complications of ERCP. Gastrointest Endosco. 2012;75(3):467-73.

3. Tenner S, Baillie J, DeWitt J, et al. American College of Gastroenterology Guideline: Management of Acute Pancreatitis. Am J Gastroenterol advance online publication, 30 July 2013.
